# Post-traumatic Stress Disorder and Its Associated Risk Factors Among Emergency Healthcare Workers: A Saudi Cross-Sectional Analytical Study

**DOI:** 10.7759/cureus.44327

**Published:** 2023-08-29

**Authors:** Faisal F Alshehri, Saleh A Alghamdi, Abdulrahman M Alrashoudi, Fahed A Albednah, Abdulrahman B Alotaibi, Abdullah M Alojayri, Amairah F Aloushan, Ghali Ahmed

**Affiliations:** 1 College of Medicine, Imam Mohammad Ibn Saud Islamic University (IMSIU), Riyadh, SAU; 2 Department of Psychiatry, College of Medicine, Imam Mohammad Ibn Saud Islamic University (IMSIU), Riyadh, SAU; 3 Department of Emergency Medicine, King Abdulaziz Medical City (KAMC) and Ministry of National Guard Health Affairs (MNGHA), Riyadh, SAU

**Keywords:** trauma, nationwide cross-sectional study, intrusive thoughts, hyperarousal, anxiety disorder, psychiatry disorders, emergency department

## Abstract

Background: Post-traumatic stress disorder (PTSD) is a psychiatric disease characterized by exposure to threatened death or serious injury and directly experiencing or witnessing the event. Many healthcare professionals have had PTSD, but emergency physicians may be particularly susceptible. To our knowledge, no study has been performed in Saudi Arabia to identify the prevalence and associated risk factors of PTSD among emergency staff.

Objective: This study aims to determine the prevalence and risk factors of post-traumatic stress disorder (PTSD) among emergency healthcare workers (HCWs) in Saudi Arabia.

Methods: A cross-sectional analytical study will be conducted in emergency departments around Saudi Arabia in all regions. The study population will include healthcare workers in emergency departments who work and presently live in Saudi Arabia. The survey was divided into two sections. The first section focuses on the emergency personnel's demographic data; the second concentrates on screening for post-traumatic stress disorder using the PTSD checklist for DSM-5 (PCL-5).

Results: Our population included 519 emergency healthcare staff, including males (51.4%) and females (48.6%). Most emergency HCWs worked in the Ministry of Health Hospitals (58%). The highest diagnosed psychological disorders among emergency staff were anxiety (19.3%) and mood disorders (10.2%). The prevalence of PTSD among emergency workers in Saudi Arabia was 14.1%. The prevalence of PTSD was significantly higher among emergency HCWs who had chronic diseases, emergency workers with anxiety or mood disorders, emergency staff who were using psychiatric medication (p<0.001), and those with psychotic disorders (p=0.002).

Conclusion: The prevalence of PTSD among emergency healthcare workers in Saudi Arabia is estimated to be 14.1%, and pre-existing mental illnesses are associated with a higher risk of PTSD.

## Introduction

Post-traumatic stress disorder (PTSD), as defined by the International Classification of Diseases 10th version (ICD-10), “Arises as a delayed or protracted response to a stressful event or situation (of either brief or long duration) of an exceptionally threatening or catastrophic nature, which is likely to cause pervasive distress in almost anyone” (2019, F43.1) [1]. The overall prevalence of PTSD varies from country to country according to multiple factors, including exposure to specific events during a lifetime. In 2003, the National Comorbidity Survey Replication (NCS-R) estimated the prevalence of PTSD among adult Americans to be (3.6%) and (9.7%) in men and women, respectively, according to DSM-IV criteria [2]. Additionally, a meta-analysis estimated that the prevalence of PTSD varies from 6.1% to 2% in mild and severe forms [3]. Many healthcare professionals have had PTSD, but emergency physicians (EP) may be particularly susceptible, owing to the fact that the nature of their work includes the possibility of frequently witnessing traumatic events and deaths, diagnostic ambiguity, high patient acuity, crowding, and circadian rhythm disruption, all of which put them at a higher risk for occupational stress and make them more susceptible to PTSD [4].

Emergency healthcare workers (HCWs) are constantly exposed to occupational trauma (such as the psychological result from encountering hazardous situations such as car accidents, fires, and major catastrophes), raising the risk of psychiatric disorders like post-traumatic stress disorder [5]. Organizations worldwide have recognized post-traumatic stress disorder among emergency service workers and given them disability insurance like the U.S. Federal Employees' Compensation Act (FECA) [6]. Those working in the emergency room (ER) are exposed to stressful events more frequently than the general population, and PTSD as a mental illness can significantly impact their lives. Certain identified factors are associated with PTSD among emergency HCWs, such as social, demographic, biological, and psychological components, morbidity, exposure to past occupational and non-occupational traumatic events, and work features, including shift work, which might cause an impact on sleep leading to a worse quality of life and an imbalance between work and social life [7,8].

Based on a single-site survey, 11.9% of emergency medicine residents in the United States fulfilled the DSM-IV diagnostic criteria for PTSD [9]. Another study in the United States recruited individuals from ten different hospitals. Overall, 12.7% of the sample tested positive for PTSD, with 33.9% having one or more clinically relevant symptoms [10]. According to recent articles, ambulance employees have a higher rate of PTSD than the general public and other emergency healthcare personnel. ER staff has a pooled PTSD prevalence of 10% compared to the general population's (1.3-3.5%) [11]. In other reports, ambulance workers globally have a greater rate of PTSD prevalence (11%) than the general population [12]. In South African ambulance personnel, the prevalence of PTSD was reported as (6.67%) and (16%) in 2005 and 2014, respectively [13,14]. Furthermore, 20% of emergency registered nurses have displayed PTSD symptoms [15]. Nationally, a study was conducted in Riyadh, Saudi Arabia, to find the risk factors related to PTSD during the COVID-19 pandemic among frontline HCWs. According to the study, the prevalence of PTSD among ER staff was 33.4%, and paramedics and nurses scored higher than physicians based on PCL-5 [16]. Moreover, a study at King Abdulaziz Medical City (KAMC) in Riyadh, Saudi Arabia, found that 26.7% of emergency healthcare providers met the diagnostic criteria for PTSD [17].

All-inclusive risk factors such as anxiety, depression, female gender, and underlying chronic illness were all indicators of high PTSD levels in physicians and nurses. Additionally, 24-hour shift work for nurses, low job experience, and poor monthly income were high-risk variables for PTSD severity [18]. To our knowledge, no study has been performed in Saudi Arabia to identify the prevalence and associated risk factors of PTSD among emergency room staff. Thus, our aims in this study are to determine the prevalence of post-traumatic stress disorder (PTSD) among emergency healthcare workers in Saudi Arabia, to assess the risk factors among healthcare workers with PTSD in the emergency department, and to find the predictors of PTSD among emergency room staff.

## Materials and methods

Study design

This is a cross-sectional analytical study that was conducted in Saudi Arabia from February 2023 to July 2023. The study population consisted of any emergency healthcare staff currently working in Saudi Arabia, such as emergency physicians, nurses, paramedics, etc. Anyone working outside the emergency department or the country was excluded. For data collection, we used a convenience technique using an online self-administered survey; we recruited data collectors voluntarily for all five regions to gather data by any means necessary, such as giving the survey face-to-face and contacting emergency healthcare providers by social media platforms through WhatsApp, Telegram, and other applications. We advised the data collectors and participants to refrain from discussing their results with each other and try to limit their interaction through social media as much as possible.

Data collection tool

The survey was divided into two sections. The first section focuses on the emergency personnel's demographic and professional information, such as sex, nationality, location, occupation, level of work, professional experience, number of shifts per month, monthly income, and chronic diseases, particularly psychiatric disorders (anxiety disorders, mood disorders, and psychotic disorders). The second section addressed screening for post-traumatic stress disorder using the PTSD checklist for DSM-5 (PCL-5).

The PCL-5 is a 20-item questionnaire corresponding to the DSM-5 PTSD symptom criteria, with the PCL-5 exhibiting high internal consistency (α=0.95). The PCL-5 symptoms are intended to encompass the four domains of PTSD (intrusive symptoms, avoidance of stimuli, negative changes in affect, and hyperarousal). The self-report rating scale for assessing the intensity of symptoms during the previous month on a five-point Likert scale of 0-4. The adjectives for the rating scale are: "Not at all," "A little bit," "Moderately," "Quite a bit," and "Extremely." The total score for Likert scale responses ranged from 0 to 80 [19]. A provisional diagnosis of PTSD has a cut-off score of 38, which has a sensitivity of 0.982 and a lower false-positive rate of 0.059 [20].

Ethical consideration

Based on the Declaration of Helsinki, Institutional Review Board approval was obtained from the Medical Research Unit, College of Medicine, Imam Mohammad ibn Saud Islamic University, Riyadh, Saudi Arabia, Project Number 398/2022, on January 1, 2023. Participation in this study was completely voluntary; each participant was notified of their consent and invited to participate. Participants did not earn a material income due to their participation.

Statistical analysis

The data were analyzed using the software program Statistical Packages for Software Sciences (SPSS) version 26 (IBM Corporation, Armonk, New York, USA). Descriptive statistics were presented using numbers and percentages (%). The relationship between PTSD and the socio-demographic characteristics of emergency HCWs has been performed using the Chi-square test. Significant results obtained from cross-tabulation were then gathered in a multivariate regression model to determine the significant independent predictor of PTSD. Values were considered significant with a p-value of less than 0.05.

## Results

Five hundred nineteen emergency HCWs were enrolled (male 51.4% vs. female 48.6%). As described in Table 1, nearly all emergency staff were Saudis (83.8%). About 38.2% were living in the central region of Saudi Arabia, with 45.7% being a physician. Most emergency HCWs worked in the Ministry of Health Hospitals (58%). About 39.7% had 13 to 16 shifts per month. Emergency HCWs who earned 10,000 to 20,000 SAR per month constitute 53.4%. Regarding the professional level, residents made up the majority of physicians surveyed (58.2%), while specialists made up the majority of specialist's professional level (83.0%) (Table 1).

**Table 1:** Socio-demographic characteristics of the emergency healthcare workers (n=519).

Study data	N (%)
Gender	
Male	267 (51.4%)
Female	252 (48.6%)
Nationality	
Saudi	435 (83.8%)
Non-Saudi	84 (16.2%)
Region of residence	
Central region	198 (38.2%)
Eastern region	70 (13.5%)
Western region	77 (14.8%)
Northern region	92 (17.7%)
Southern region	82 (15.8%)
Professional status	
Physician	237 (45.7%)
Nurse	166 (32.0%)
Paramedics	80 (15.4%)
Respiratory therapist	19 (03.7%)
Other allied position	17 (03.3%)
Type of hospital	
Ministry of Health Hospitals	301 (58.0%)
Military hospitals	68 (13.1%)
University hospitals	78 (15.0%)
Private hospitals	39 (07.5%)
Saudi Red Crescent Authority	33 (06.4%)
Number of shifts per month	
≤12	125 (24.1%)
13–16	206 (39.7%)
>16	188 (36.2%)
Monthly income (SAR)	
<10,000	169 (32.6%)
10,000–20,000	277 (53.4%)
>20,000	73 (14.1%)
Physician’s professional level (n=237)	
Consultant	19 (8.0%)
Fellow	13 (5.5%)
Registrar	28 (11.8%)
Resident	138 (58.2%)
Intern	39 (16.5%)
Specialist’s professional level (n=282)	
Specialist consultant	08 (02.8%)
Specialist first class	28 (09.9%)
Specialist	234 (83.0%)
Other	12 (04.3%)

**Figure 1:**
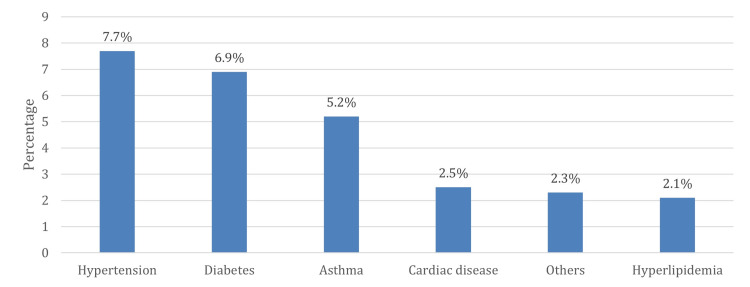
Prevalence of chronic diseases among emergency staff.

The highest reported psychological disorders among emergency staff were anxiety (19.3%) and mood disorders (11.1%), and the most common type of anxiety disorder was generalized anxiety disorder (GAD 7.3%). In comparison, the most prevalent mood disorder was major depressive disorder (9.2%). Most of the respondents did not report psychotic disorders (95%). The most commonly used psychiatric medication was antidepressant medication (7.7%) (Table 2). Figure 1 shows the prevalence of chronic diseases among emergency staff. The most common chronic diseases among emergency HCWs were hypertension (7.7%) and diabetes (6.9%).

**Table 2:** Prevalence of psychological disorders among emergency HCWs and psychiatric medication use (n=519). SSRIs: selective serotonin reuptake inhibitors; SNRI: serotonin and norepinephrine reuptake inhibitors, HCWs: healthcare workers.

Variables	N (%)
Anxiety disorder	
None	419 (80.7%)
Generalized anxiety disorder	38 (07.3%)
Social phobia	20 (03.9%)
Panic disorder	23 (04.4%)
Other	19 (03.7%)
Mood disorder	
None	461 (88.8%)
Major depressive disorder	48 (09.2%)
Bipolar disorder	10 (01.9%)
Psychotic disorder	
None	493 (95.0%)
Schizophrenia	06 (01.2%)
Schizoaffective disorder	06 (01.2%)
Delusional disorder	14 (02.7%)
Use of psychiatric medication	
None	451 (86.9%)
Antidepressant medication (e.g., SSRI, SNRI, etc.)	40 (07.7%)
Mood stabilizer medications (e.g., valproic acid, lithium, carbamazepine, etc.)	07 (01.3%)
Benzodiazepine (e.g., Xanax, Rivotril, Valium, Ativan, etc.)	07 (01.3%)
Antipsychotic medications (e.g., Quetiapine, Olanzapine, etc.)	06 (01.2%)
Other	08 (01.5%)

**Figure 2:**
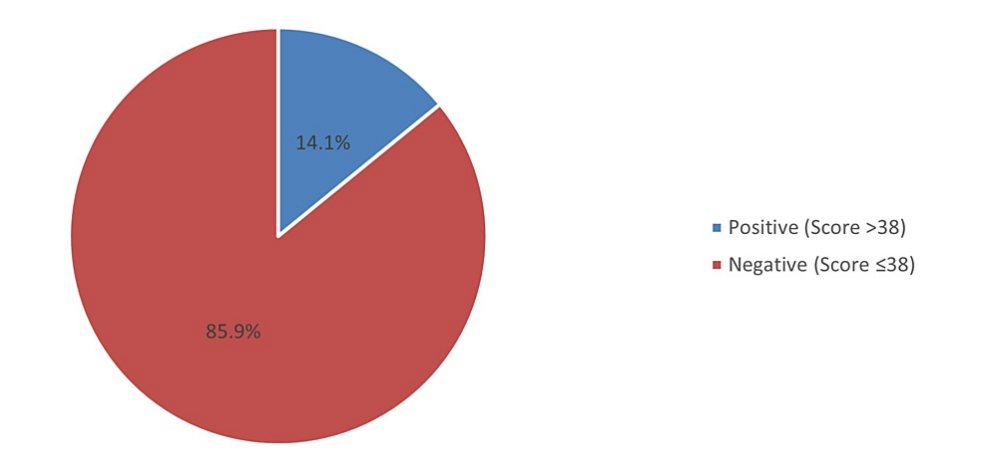
Prevalence of PTSD among emergency healthcare workers PTSD: post-traumatic stress disorder.

Figure 2 displays the prevalence of PTSD among emergency HCWs. Based on the PCL-5 questionnaire, the prevalence of PTSD among emergency HCWs was 14.1%. The most debilitating reported symptoms in the PCL-5 questionnaire that affected emergency HCWs were trouble falling or staying asleep (mean score: 1.34), followed by loss of interest in activities (mean score: 1.24) and difficulty concentrating (mean score: 1.21), while the least of them was having strong physical reactions (mean score: 0.82) (Table 3).

**Table 3:** Assessment of PTSD according to PCL-5 (n=519). Response has a range from 0=“not at all” to 4=“always”. PTSD: post-traumatic stress disorder.

In the past month, how much were you bothered by	Mean ± SD	Rank
Repeated, disturbing, and unwanted memories of the stressful experience?	1.16 ± 1.09	6
Repeated, disturbing dreams of the stressful experience?	0.97 ± 1.09	18
Suddenly feeling or acting as if the stressful experience were actually happening again (as if you were actually back there reliving it)?	0.98 ± 1.09	16
Feeling very upset when something reminded you of a stressful experience?	1.16 ± 1.10	5
Having strong physical reactions when something reminded you of the stressful experience (for example, heart pounding, trouble breathing, sweating)?	0.82 ± 1.06	20
Avoiding memories, thoughts, or feelings related to the stressful experience?	1.13 ± 1.14	7
Avoiding external reminders of the stressful experience (for example, people, places, conversations, activities, objects, or situations)?	1.00 ± 1.10	14
Trouble remembering important parts of the stressful experience?	0.97 ± 1.09	17
Having strong negative beliefs about yourself, other people, or the world (for example, having thoughts such as: I am bad, there is something seriously wrong with me, no one can be trusted, the world is completely dangerous)?	1.05 ± 1.14	9
Blaming yourself or someone else for the stressful experience or what happened after it?	1.03 ± 1.09	10
Having strong negative feelings such as fear, horror, anger, guilt, or shame?	1.11 ± 1.15	8
Loss of interest in activities that you used to enjoy?	1.24 ± 1.24	2
Feeling distant or cut off from other people?	1.20 ± 1.22	4
Trouble experiencing positive feelings (for example, being unable to feel happiness or have loving feelings for people close to you)?	1.02 ± 1.18	11
Irritable behavior, angry outbursts, or acting aggressively?	1.00 ± 1.14	13
Taking too many risks or doing things that could cause you harm?	0.86 ± 1.11	19
Being “superalert” or watchful or on guard?	1.04 ± 1.14	12
Feeling jumpy or easily startled?	0.99 ± 1.14	15
Having difficulty concentrating?	1.21 ± 1.22	3
Trouble falling or staying asleep?	1.34 ± 1.26	1

Table 4 demonstrates the relationship between the level of PTSD with socio-demographic characteristics and other psychological disorders. It was discovered that the prevalence of PTSD was significantly higher among emergency HCWs who had chronic diseases (p<0.001), also emergency workers with anxiety or mood disorders (p<0.001), and those with psychotic disorders (p=0.002), and emergency staff who were using psychiatric medication (p<0.001). No significant differences were observed between PTSD and participants' socio-demographic data (p>0.05).

**Table 4:** Relationship between PTSD according to the socio-demographic characteristics and the prevalence of psychological disorders in emergency healthcare workers (n=519). §P-value has been calculated using the Chi-square test. **Significant at p<0.05 level. PTSD: post-traumatic stress disorder.

Factor	PTSD		P-value^§^
	Positive N (%) (n=73)	Negative N (%) (n=446)	
Gender			
Male	32 (43.8%)	235 (52.7%)	0.161
Female	41 (56.2%)	211 (47.3%)	
Nationality			
Saudi	63 (86.3%)	372 (83.4%)	0.534
Non-Saudi	10 (13.7%)	74 (16.6%)	
Region of residence			
Central region	24 (32.9%)	174 (39.0%)	0.518
Eastern region	13 (17.8%)	57 (12.8%)	
Western region	14 (19.2%)	63 (14.1%)	
Northern region	11 (15.1%)	81 (18.2%)	
Southern region	11 (15.1%)	71 (15.9%)	
Professional status			
Physician	29 (39.7%)	208 (46.6%)	0.393
Nurse	23 (31.5%)	143 (32.1%)	
Paramedics	16 (21.9%)	64 (14.3%)	
Other allied position	05 (06.8%)	31 (07.0%)	
Type of hospital			
Ministry of Health Hospitals	43 (58.9%)	258 (57.8%)	0.571
Military hospitals	12 (16.4%)	56 (12.6%)	
University hospitals	07 (09.6%)	71 (15.9%)	
Private hospitals	05 (06.8%)	34 (07.6%)	
Saudi Red Crescent Authority	06 (08.2%)	27 (06.1%)	
Number of shifts per month			
<13	16 (21.9%)	109 (24.4%)	0.844
13–16	31 (42.5%)	175 (39.2%)	
>16	26 (35.6%)	162 (36.3%)	
Monthly income (SAR)			
<10,000	28 (38.4%)	141 (31.6%)	0.455
10,000–20,000	37 (50.7%)	240 (53.8%)	
>20,000	08 (11.0%)	65 (14.6%)	
Associated chronic disease			
No	50 (68.5%)	363 (81.4%)	0.011**
Yes	23 (31.5%)	83 (18.6%)	
Anxiety disorder			
No	40 (54.8%)	379 (85.0%)	<0.001**
Yes	33 (45.2%)	67 (15.0%)	
Mood disorder			
No	52 (71.2%)	409 (91.7%)	<0.001**
Yes	21 (28.8%)	37 (08.3%)	
Psychotic disorder			
No	64 (87.7%)	429 (96.2%)	0.002**
Yes	09 (12.3%)	17 (03.8%)	
Use of psychiatric medication			
No	47 (64.4%)	404 (90.6%)	<0.001**
Yes	26 (35.6%)	42 (09.4%)	

A multivariate regression analysis was conducted, revealing that having anxiety and using psychiatric medication were significant independent predictors of PTSD in emergency staff. This further suggests that compared to emergency HCWs without anxiety disorders, emergency HCWs with anxiety disorders have higher chances of PTSD by at least 2.8 times higher (AOR=2.796; 95% CI=1.517-5.151; p=0.001). Emergency HCWs using psychiatric medication had an increased risk of developing PTSD by at least 2.6-fold higher than emergency HCWs who were not using it (AOR=2.623; 95% CI=1.172-5.874; p=0.019). Variables such as associated chronic disease, mood disorders, and psychotic disorders were not relevant predictors of PTSD after adjustment to a regression model (p>0.05) (Table 5).

**Table 5:** Multivariate regression analysis for the predictors of PTSD among emergency healthcare workers (n=519). AOR: adjusted odds ratio; CI: confidence interval; PTSD: post-traumatic stress disorder. **Significant at p<0.05 level.

Factor	AOR	95% CI	P-value
Associated chronic disease			
No	Ref		
Yes	1.290	0.683–2.436	0.432
Anxiety disorder			
No	Ref		
Yes	2.796	1.517–5.151	0.001**
Mood disorder			
No	Ref		
Yes	1.613	0.751–3.465	0.220
Psychotic disorder			
No	Ref		
Yes	0.793	0.268–2.347	0.675
Use of psychiatric medication			
No	Ref		
Yes	2.623	1.172–5.874	0.019**

## Discussion

This study aims to estimate the prevalence of PTSD among emergency workers and its associated risks. The overall prevalence of PTSD among emergency workers in Saudi Arabia is estimated to be 14.1%, based on our findings. This finding signifies that emergency workers are at more risk than the average individual, estimated at 6.8% [2]. Moreover, in 2020, a rapid systematic review study assessed the prevalence of PTSD among emergency department staff, and it was 18.6%, which is slightly higher than our result, confirming that emergency staff is a vulnerable group to develop PTSD [21]. Furthermore, the estimated prevalence among emergency physicians was 12.24 percent, close to the estimated prevalence of PTSD by other studies in Germany, Pakistan, and Belgium [22-24]. Additionally, female emergency workers had a higher rate of PTSD than males (56.2% vs. 43.8%). This finding is supported by a 2022 study in which 57.4% of females and 42.6% of males had PTSD, respectively [17]. Nurses have the highest percentage of PTSD among emergency staff (13.86%), followed by physicians (12.24%). Similarly, another study found that nurses are at much higher risk than physicians at 25.8% and 15.6%, and in the nurse's group, the female gender was higher than males [21]. Emergency service providers encounter a variety of stressors while performing their duties, such as life-threatening scenarios and deeply upsetting images of disaster [13]. We found that the estimated risk of developing PTSD in emergency services was 20%. Whereas another study estimated a risk of 6.67% less than ours [13].

Work-related stress is any adverse physical or psychological reaction to overwhelming job requirements that exceed the worker's ability to adapt [25]. According to the American Psychological Association, being constantly exposed to work-related stress has adverse effects on individuals' mental and physical well-being and can contribute to different health disorders. Emergency professionals are subjected to high-stress working environments as they must provide critical care for patients; their inability to provide quality care can sometimes have a negative psychological impact, increasing the risk of psychiatric and medical illnesses [5,25]. Based on our study, the highest reported psychiatric disorders among emergency staff were anxiety (19.3%) and mood disorders (11.1%), and the most common type of anxiety disorder was generalized anxiety disorder. In comparison, the most prevalent mood disorder was major depressive disorder. The incidence of PTSD and depression in the present sample is in line with previous work of European samples [26]. The prevalence of PTSD was significantly higher among emergency HCWs with chronic diseases, anxiety or mood disorders, psychotic disorders, and emergency staff using psychiatric medication. This aligns with previous findings that pre-existing mental illnesses are associated with a higher risk of PTSD among healthcare workers, including those in the emergency department [27]. In addition, mounting evidence exists that among healthcare professionals, anxiety disorders, major depressive disorder, and PTSD impair patient outcomes and raise the incidence of medical errors [28,29]. Our bivariate analysis showed no statistically significant difference between the mean PTSD severity score by gender, marital status, professional status, type of hospital, number of shifts per month, and monthly income, which is consistent with the literature [4,28].

Underlying mental health issues, coexisting illnesses, a history of child abuse, a higher degree of acute stress symptoms, and a more severe traumatic event as the incident are risk factors for developing PTSD in the general population [30,31]. In newly trained paramedics, psychological characteristics like neuroticism and dissociation, the use of unhealthy coping mechanisms like disengagement, and cognitive features like self-perceived resilience and emotion suppression predicted a higher chance of developing PTSD [26]. Workplace violence, bullying, the loss of a child, the stress of litigation, the use of electronic health records, long work hours, and circadian disruption brought on by night shifts are all potential risk factors for PTSD in the healthcare industry, especially for emergency staff [28,29,32].

Likewise, chronic medical diseases have been shown to be a contributing risk factor for developing PTSD in the general population after disturbing events [31]. Our study found that the prevalence of PTSD was significantly higher among emergency HCWs suffering from chronic medical conditions like hypertension and diabetes; as such, these are the most prevalent chronic medical diseases associated with PTSD in our population. Correspondingly, our results correlate with a previous finding showing that HCWs in the emergency department with underlying chronic diseases were more susceptible to developing high PTSD severity [18].

Our findings suggest that the most exhausting symptoms reported by emergency staff on the PTSD assessment scale were trouble falling asleep and loss of interest in activities with difficulty concentrating. On the contrary, a study conducted in the United States among emergency physicians found that the most commonly reported symptoms are feeling distant or cut off from other people, having trouble falling or staying asleep, and having strong negative beliefs about yourself or the world [33]. These results suggest the need for intervention to control the occupational stressors in the emergency room as it can impair work performance, causing a high rate of absenteeism and presenteeism and, more importantly, impair the ability of decision-making that might impact the provided healthcare service and patient outcomes negatively.

Protective factors may deepen or buffer the effects of PTSD among ER staff. Knowing which risk factors are significantly related to PTSD would help guide any interventional program and optimize PTSD management. A multivariate regression model was used to determine which variables were uniquely predictive of PTSD among ER staff. As observed from our questionnaire, having a baseline anxiety disorder was a statistically significant predictor of PTSD. This finding is supported by prior studies established among emergency healthcare providers. Anxiety has been identified as a risk factor for PTSD; those who reported higher anxiety levels were more likely to acquire PTSD symptoms after witnessing traumatic occurrences [11]. A prior study found anxiety to be a predictor of PTSD in other populations, such as military people and natural catastrophe survivors [34]. Alongside, ER staff using psychiatric medication were found to be more prone to PTSD than others. The remaining factors, including mood disorders, psychotic illnesses, diabetes, and hypertension, were not significant as PTSD predictors, according to our multivariate analysis.

Our study has some limitations that should be acknowledged. Cross-sectional studies using convenient sampling techniques that rely on online self-reported data have a risk of bias, even though our study used a PCL-5-validated questionnaire for screening PTSD. However, PCL-5 is a screening tool for PTSD that can assist in reaching a diagnosis, but the proper diagnosis should be clinician-based through clinical assessment. Because of the nature of our population, reaching a higher sample was difficult. Due to their knowledge, emergency health providers may under-report or over-report their symptoms depending on factors such as social desirability, bias, or fear of stigma. Cross-sectional studies cannot frequently show causality or the temporal correlation between variables; emergency healthcare workers who have had signs and symptoms of PTSD for a prolonged period might influence the outcomes. Moreover, it does not consider variations in PTSD symptoms over time. Emergency healthcare providers may be exposed to varying amounts of stress and trauma at different periods in their careers, and their symptoms may fluctuate correspondingly.

## Conclusions

The overall prevalence of PTSD among emergency healthcare workers in Saudi Arabia is estimated to be 14.1%, with female emergency healthcare workers having a higher rate of PTSD than males. Pre-existing medical and mental illnesses, such as anxiety and mood disorders, are associated with a higher risk of PTSD. In future studies, we urge methods to reduce the risk and provide optimal management of PTSD among ER workers. Organizational and workplace predictors should be thoroughly addressed; managing other medical and mental illnesses in emergency healthcare workers, improving the work environment, and regulating physical and emotional stressors, will help reduce the risk of PTSD among healthcare staff in the emergency department.
